# Emerging functions of Plakophilin 4 in the control of cell contact dynamics

**DOI:** 10.1186/s12964-025-02106-1

**Published:** 2025-02-25

**Authors:** Lisa Müller, Mechthild Hatzfeld

**Affiliations:** 1 Institute of Molecular Medicine, Section for RNA biology and Pathogenesis, Martin Luther University Halle-Wittenberg, Charles Tanford Protein Research Center, Kurt-Mothes-Str. 3A, 06120 Halle, Germany; 2 Institute of Molecular Medicine, Section for Pathobiochemistry, Martin Luther University Halle-Wittenberg, Charles Tanford Protein Research Center, Kurt-Mothes-Str. 3A, 06120 Halle, Germany

**Keywords:** Plakophilin 4 (p0071), Adherens junctions, Keratinocytes, Rho signaling, GEFs, GAPs, Cell mechanics, Hippo pathway, YAP/TAZ, Cell adhesion

## Abstract

Plakophilin 4 (PKP4, also called p0071) is a unique armadillo family protein localized at adherens junctions that acts as a scaffold protein capable of clustering cadherins. PKP4 also regulates cadherin recycling which is vital to enable junction dynamics. In addition, PKP4 controls the mechanical properties of cells by regulating actin filament organization through small Rho-GTPases. In this setting, PKP4 controls the localization and activity of specific guanine exchange factors (GEFs) and of their opponents, the GTPase activating proteins (GAPs). Through the formation of multiprotein complexes with Rho-GTPases, their regulators and their effectors, PKP4 controls the spatio-temporal activity of Rho signaling to regulate cell adhesion and cell mechanics. In keratinocytes, PKP4 prevents differentiation and at the same time dampens proliferation. This is, in part achieved through an interaction with the Hippo pathway, which controls the activity of the transcriptional co-factors YAP and TAZ. In a feedback loop, YAP/TAZ modulate PKP4 localization and function. Here, we review the various functions of PKP4 in cell signaling, cell mechanics, cell adhesion and growth control. We discuss how these functions converge in the regulation of cell adhesion dynamics to allow cells to adapt to their changing environment and enable proliferation, delamination but, at the same time, guarantee cell barrier function.

## Background

Plakophilin 4 (PKP4, also called p0071) was first described as an armadillo family protein related to p120-catenin (p120ctn/CTNND1) [1]. PKP4 localized predominantly at cell-cell borders and was identified as a component of adhesion plaques. Like other armadillo family proteins, it also localizes in the cytoplasm and in the nucleus suggesting that it functions not only as a structural protein of adherens junctions (AJs) but also in cell signaling. PKP4 is most closely related to δ-catenin (CTNND2) which is exclusively expressed in neural and neuroendocrine tissues. Hence, this protein was called neural plakophilin-related arm-repeat protein (NPRAP) [2]. Armadillo repeat gene deleted in Velo-cardio-facial syndrome (VCFS), another member of this protein family, was isolated from the deleted region in VCFS and called ARVCF (armadillo repeat gene deleted in VCFS) [3, 4]. ARVCF associated with E-cadherin and competed with p120ctn for interaction with the E-cadherin juxtamembrane domain. Despite ubiquitous expression, ARVCF is less abundant than p120ctn in most cell types [4]. PKP4, p120ctn, NPRAP and ARVCF share functions in modulating the turnover rate of membrane-bound cadherins and in regulating the activity of small Rho GTPases.

**Fig. 1:**
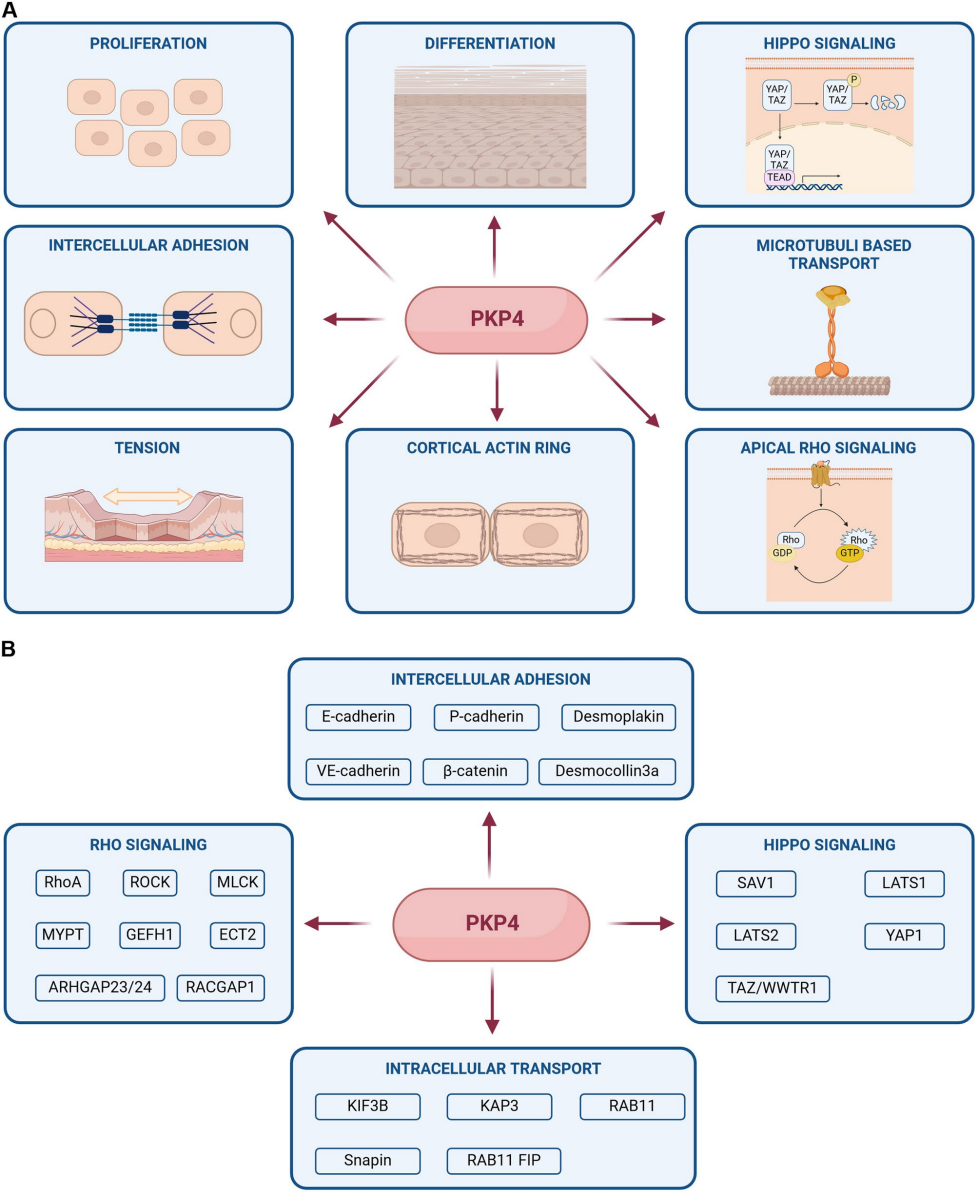
Overview about PKP4 functions and protein interactions. (**A**) Schematic depicting the various cellular processes regulated by PKP4. (**B**) Summary of PKP4 protein interactions involved in intercellular adhesion, intracellular transport, Rho- and Hippo signaling

Here, we focus on PKP4 and summarize its functions as a variable scaffold protein in regulating intercellular adhesion, actin organization and generation of tissue tension, proliferation and differentiation through its participation in various signaling processes mediated by multiple protein-protein interactions (Fig. 1A, B). We show that these diverse functions converge to regulate and adapt intercellular adhesion to changing requirements depending on the cellular environment.

### Expression of PKP4

PKP4 is widely expressed with the highest mRNA levels in the brain and kidney and lower expression in all other organs (https://www.proteinatlas.org/ENSG00000144283-PKP4/tissue). It localizes primarily at the intracellular plaques of AJs of diverse epithelia and some endothelia, as well as in the area composita of the intercalated disks of cardiomyocytes [5]. It was prominently detected in kidney tubules [6]. In the skin, its expression is restricted to the basal proliferative layer and declines with keratinocyte differentiation in the suprabasal layers. Protein expression also declines in cultured keratinocytes when these cells are induced to differentiate [7] suggesting a differentiation specific role in this tissue.

### Function of PKP4 in cell adhesion

PKP4 localizes at cell borders and its relationship to p120ctn suggested a role in AJ where p120ctn binds to the juxtamembrane region of cadherin cytoplasmic tails and supports cadherin clustering [8]. The recruitment of p120ctn to junctions depended on its interaction with classical cadherins. Moreover, p120ctn was required for the transition from weak to strong cell-cell adhesion mediated by cadherin clustering. A recent computational study shows that p120ctn could allosterically regulate the cis-dimerization of cadherins through two mechanisms: (1) Promotion of cadherin clustering by oligomerization; (2) Increase in lateral interactions between cadherins on the cell surface [9].

PKP4 localizes along cell borders, co-localizes with classical cadherins and its overexpression induced cadherin recruitment to the plasma membrane [10, 11]. High-resolution immunofluorescence microscopy and immunoelectron microscopy demonstrated a specific localization at AJs. Immunoprecipitation experiments suggested an association with E-cadherin and β-catenin but not with desmoglein 1 and 2 [5]. Other studies described a partial overlap with desmosomal proteins [10, 12]. A closer analysis of protein interactions revealed that the central armadillo repeat domain associated with AJs and enhanced membrane association of classical cadherins, whereas the head domain of PKP4 was sufficient for desmosomal targeting. The tail domain localized preferentially to the nucleus. In agreement with localization studies, the central armadillo repeat domain bound to various classical cadherins whereas the head domain interacted with the desmosomal proteins desmocollin 3a and desmoplakin. Thus, PKP4 could interact with desmosomal and AJ proteins at the same time through the different protein domains although this remains to be directly demonstrated. The exact nature and function of PKP4’s interactions with desmosomal proteins, how it might depend on cell density, cell signaling and/or protein modifications awaits future studies. Overexpression of wildtype (WT) PKP4 enhanced E-cadherin membrane recruitment and AJ assembly but dramatically compromised desmosome assembly, resulting in keratin filament retraction from regions of cell-cell contact [10, 11]. This suggests that PKP4 plays a role in coordinating the balance between AJs and desmosomes: high expression of PKP4 would result in a preponderance of AJs over desmosomes whereas a decline in its expression as observed e.g. upon keratinocyte differentiation would favor desmosomes assembly. Moreover, the preponderance of AJs compared to desmosomes in PKP4 expressing cells is compatible with weaker adhesion that enables dynamic remodeling of tissues.

PKP4 is also present at dermal microvascular endothelial intercellular junctions and colocalized with VE-cadherin, an endothelium-specific cadherin [12]. As expected, PKP4 directly bound VE-cadherin and competed with p120ctn for binding to the juxtamembrane region in the cytoplasmic tail of VE-cadherin. Accordingly, overexpression of PKP4 displaced p120ctn from intercellular junctions. Again, desmoplakin was found to associate with the non-armadillo head domain of PKP4 suggesting that PKP4 might play a role in balancing AJ- versus desmosome-mediated adhesion in endothelial as well as epithelial cells [12].

### Function of PKP4 in vesicle recycling

**Fig. 2:**
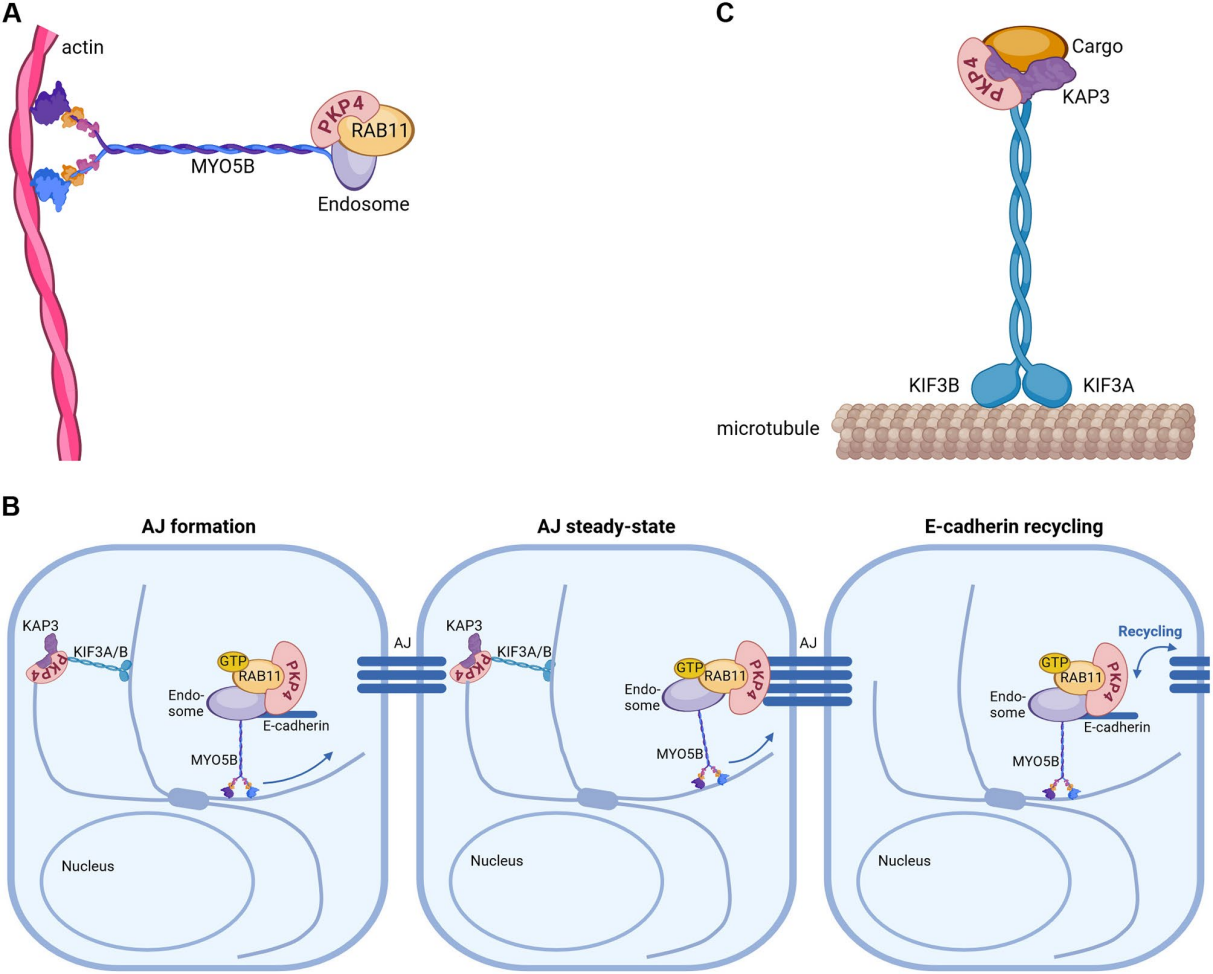
PKP4 functions in intracellular transport and recycling. (**A**) PKP4 interacts with Rab11-expressing endosomes to promote myosin-Vb (MYO5B)-mediated transport. (**B**) Schematic depicting the role of PKP4 in mediating E-cadherin transport and recycling during AJ formation, at steady state and during recycling. (**C**) PKP4 stabilizes the interaction of KIF3 motor proteins and the cargo adapter KAP3

Localization and stabilization of cadherins at the plasma membrane requires not only a function in cadherin clustering but also depends on their transport to the membrane. Small Rab GTPases are key regulators of intracellular membrane trafficking. They define vesicle identity and ensure that cargo proteins are delivered to their correct destinations at the right time. Rab4, Rab5 and Rab11 are involved in vesicle recycling [13–15]. Endocytosed proteins are first delivered to sorting endosomes from where they either recycle back to the plasma membrane or are delivered to lysosomes for degradation. Rab4 mediates recycling directly from early endosomes to the plasma membrane whereas Rab11 passes proteins to recycling endosomes before they are transported back to the cell surface [16, 17]. Rab11 is a key regulator of the surface expression of receptors and adhesion proteins [17, 18]. It is involved in the transport and sorting of newly synthesized as well as endocytosed E-cadherin by recycling endosomes [19–22] thereby regulating cadherin-mediated intercellular adhesion. PKP4 interacts directly with Rab11a (Fig. 2A) and controls the localization and recycling of Rab11a but is also recycled through the Rab11-dependent route. Thus, Rab11a regulates intercellular adhesion by controlling PKP4 and E-cadherin recycling (Fig. 2B). In turn, PKP4 contributes to the regulation of Rab11 localization and links recycling to AJ formation to allow the dynamic modulation of intercellular adhesion as required during tissue remodeling, proliferation and migration [23].

Kinesin superfamily proteins (KIFs) are motor proteins that transport organelles and macromolecules along microtubules. The kinesin superfamily protein 3 (KIF3) is a heterotrimeric complex that consists of two kinesin motor proteins, KIF3A and KIF3B, and a cargo adaptor subunit, KAP3 (also known as KIFAP3). KAP3 has been implicated in weakening RhoA-mediated cell-cell adhesion, in transporting N-cadherin and in impairing basement membrane formation [24, 25]. PKP4 interacts directly with KIF3B and KAP3 and stabilizes the association between KIF3B and the KAP3 cargo-binding subunit (Fig. 2C). Moreover, PKP4 mediates directional vesicle movement along microtubules and secretion of different KIF3-transported cargo [26]. In addition, the KIF3 complex controls PKP4 localization during mitosis [27]. Cytokinesis depends on coordinated membrane trafficking to deliver new membrane to the furrow to complete abscission. Rab11- and FIP3-containing recycling endosomes accumulate near the cleavage furrow and are required for successful completion of cytokinesis and abscission [28]. Thus, PKP4 coordinates motor transport to the contractile ring leading to the accumulation of recycling endosomes to allow for membrane expansion and finally abscission.

### Function of PKP4 in actin filament organization and the generation of tissue tension

A hallmark of AJs is their association with a submembranous actin cytoskeleton. The cadherin-catenin system binds to cortical actin filaments and these actin networks can associate with non-muscle myosin II to generate tension and modulate mechanical properties. The tight control of actin filament turnover and organization together with myosin-II activity regulates mechanical forces that drive the assembly, maintenance and remodeling of AJs [29, 30]. Actin polymerization is required for the formation of AJs whereas their stabilization and maturation critically depends on actomyosin tension. Actin network organization and myosin-II-mediated tension are crucial for tethering E-cadherin in the plasma membrane [29].

**Fig. 3:**
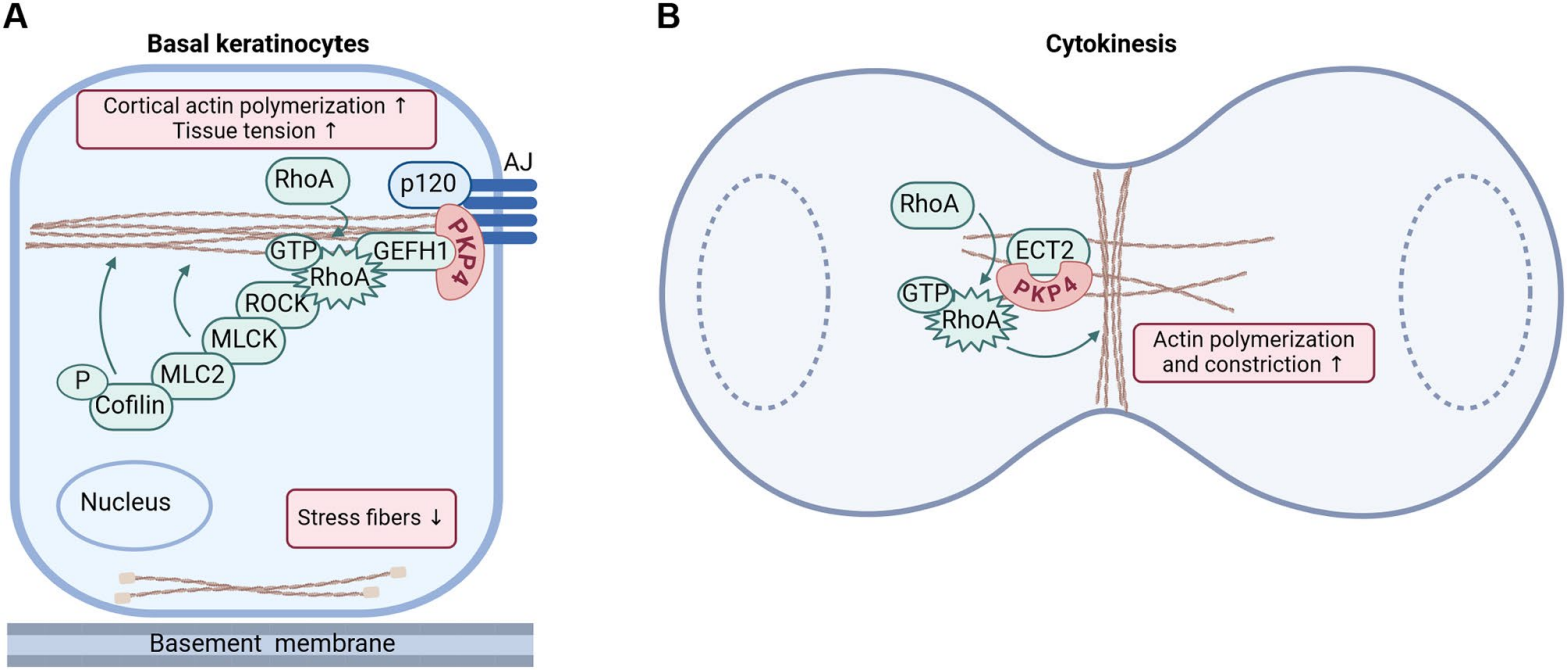
PKP4 functions in the local control of Rho activity. (**A**) Schematic depicting the role of PKP4 in supporting a GEFH1-RhoA-ROCK-MLC signaling axis to promote cortical actin ring formation and actomyosin-generated tension in basal keratinocytes. (**B**) During cytokinesis, PKP4 facilitates Ect2-mediated local RhoA activation at the cleavage furrow to enable abscission of the two daughter cells

The dynamic connection of AJs to the actin cytoskeleton is tightly controlled by small GTPases, such as RhoA and Rac which localize at AJs and regulate cell mechanics by modulating the actin cytoskeleton [30]. When misregulated, abnormal junctional actin polymerization can drive pathological conditions including cancer, vascular and neurodegenerative diseases [31]. RhoA activates several downstream effectors, such as Rho-associated kinases 1 and 2 (ROCK1 and ROCK2) that phosphorylate the myosin regulatory light chains (MLC), leading to activation of myosin and generation of actomyosin contractile forces. ROCK also activates LIM-kinase (LIMK) which inhibits cofilin’s actin depolymerization function, thus stabilizing filamentous actin (F-actin). ROCK1 depletion resulted in the mislocalization of the cadherin complex and of cortical actin [32] and ROCK was necessary to stabilize GTP-RhoA and sustain junctional tension [33]. Junction formation and stability as well as the generation of intrinsic forces depend on the precise spatio-temporal regulation of RhoA activity and specific RhoA effectors. In murine keratinocytes, PKP4 promoted the formation of the cortical actin ring and suppressed stress fiber formation [34]. This was achieved by an association of PKP4 with RhoA regulators as well as RhoA effectors to locally control RhoA activity and thus contractility at the cell cortex. These data suggest that AJ-associated PKP4 provides a scaffold for the Rho activator ARHGEF2 (GEFH1) and the RhoA effectors myosin light chain kinase (MLCK) and MLC2, thereby promoting the spatio-temporal activation of RhoA signaling at cell junctions to allow cortical actin ring formation and actomyosin contraction [34] (Fig. 3A). In support, PKP4 depletion resulted in a complete loss of intrinsic tension in murine keratinocytes. The formation and stability of the circumferential actin belt was further supported by an association of PKP4 with the RhoA suppressor ARHGAP23, which prevented RhoA activation in the cytoplasm thereby limiting stress fiber formation [34]. Active RhoA is commonly pulsatile in medial-apical regions but sustained at AJs [33]. Considering the role of PKP4 in generating and supporting junctional tension, one might speculate that PKP4 maintains the spatiotemporal dynamics of RhoA and myosin II-based contractility at AJs.

Some PDZ domain containing proteins such as Scribble or ZO1 strengthen the cell adhesion machinery at AJs and tight junctions (TJs) upon loading forces to preserve epithelial homeostasis. PKP4, in contrast to p120ctn, has a PDZ binding domain at its C-terminus through which it interacts with Scribble [35] and other PDZ proteins (e.g. erbin, papin) [36, 37] (https://thebiogrid.org/114074). Thus, one function of PKP4 could be to recruit such proteins to the junctional zone once tension has been generated. In agreement, loss of PKP4 interfered with junction maturation and stability in murine keratinocytes [34]. Taken together, these data identify PKP4 as an AJ component that transduces mechanical signals into cytoskeletal organization and junction maturation.

PKP4 controls Rho signaling not only at the AJs but also during mitosis [27, 38, 39] (Fig. 3B). During cytokinesis, PKP4 regulates the local activation of RhoA without affecting global Rac1 or Cdc42 activities. RhoA-dependent contractile ring formation required for abscission was disturbed after PKP4 depletion resulting in failed cytokinesis and multinucleation [38]. After being transported to the region of ingression via KIF3, PKP4 induced RhoA activation required for contractile ring formation and abscission. Here, PKP4 associated not only with RhoA but also with the GEF epithelial cell transforming 2 (Ect2) and both, PKP4 and Ect2 were required for full activation of RhoA in vitro [38, 39].

PKP4 is also involved in actin remodeling in mouse embryonic fibroblasts and in neuronal cells [40]. Fragile X mental retardation protein (FMRP) is a widely expressed RNA-binding protein that functions as a translational repressor. Its activity is essential for proper synaptic plasticity and architecture. Loss of FMRP expression leads to the Fragile X Syndrome (FXS), a neurodevelopmental disorder characterized by intellectual disability and behavioral symptoms. This correlates with the dysregulation of actin dynamics leading to alterations in dendritic spine morphology and synaptic plasticity, in the development of dendrites and axons and in underlying learning and memory [41, 42]. In order to elucidate the molecular mechanism, hundreds of FMRP-target mRNAs were identified but few interactions between FMRP and actin-regulating proteins have been validated. Our group has identified the PKP4 mRNA as a FMRP target. FMRP interfered with the translation of the PKP4 mRNA in a 3’-UTR-dependent manner. Accordingly, FMRP-depleted cells revealed elevated levels of PKP4 protein. In mouse fibroblasts lacking FMRP, the actin cytoskeleton was markedly reorganized with reduced stress fibers compared to fibroblasts expressing FMRP. A knockdown of PKP4 rescued this phenotype. In neuronal cells, elevated PKP4 expression in the FMRP free cells increased dendritic branching, but not length. Again, a knockdown of PKP4 decreased dendritic branching and length and thus rescued the phenotype. In contrast, overexpression of PKP4 mimicked the effects of FMRP depletion. Thus, PKP4 and FMRP regulated neurite outgrowth and branching in a diametrically opposed way in agreement with the role of FMRP as a negative regulator of PKP4 [40]. These data identified PKP4 as an important FMRP target and strongly suggest that impaired actin cytoskeletal functions mediated by an excess of PKP4 are a key aspect underlying the fragile X syndrome [40].

Similar alterations of the neuronal actin cytoskeleton occur in a variety of neurological disorders including schizophrenia and several forms of autism spectrum disorders. Interestingly, PKP4 has been implicated in these diseases based on genome-wide expression profiling [43–45] supporting an important role of PKP4 in actin organization in the nervous system.

The turnover of AJs components is achieved by endocytosis and recycling of cadherins to the cell surface [46]. Several studies point to a role of actin in mediating E-cadherin endocytosis and E-cadherin recycling is important for AJs dynamics [47]. However, association with actin has also been proposed to stabilize E-cadherin in AJs [48]. The involvement of PKP4 in actin based AJ stabilization and in recycling is compatible with a role in balancing these two processes to allow dynamic adaptation to environmental changes and stress conditions. Future studies are required to elucidate how PKP4 switches between cadherin stabilization and recycling and the respective interaction partners. Given the numerous phosphorylation site in PKP4, PKP4 functions may be regulated by several signaling pathways through site-specific phosphorylation.

### Function of PKP4 in growth control and differentiation

#### PKP4 in proliferation and differentiation

In the epidermis, PKP4 expression is restricted to the basal proliferative layer, which correlates with a precursor cell state. In contrast to prediction, PKP4 depletion promoted keratinocyte proliferation and resulted in cells that were smaller than WT keratinocytes. The decrease of cells in G0/G1 phase indicates that these cells proceed faster through the cell cycle [7]. Proliferation and differentiation are typically inversely correlated in keratinocytes and differentiation in the suprabasal layers correlates with a loss of the proliferative potential. However, differentiation and proliferation although regulated simultaneously are independent and cells often start differentiating before they stop dividing to allow expansion of cell numbers and acquisition of differentiated function to occur in parallel [49]. Loss of PKP4 correlated with the premature expression of epidermal differentiation markers including loricrin, filaggrin, and keratins 1 and 10 indicating that PKP4 expression in the basal cell layer of the epidermis prevents differentiation. Increased differentiation in PKP4-depleted keratinocytes also facilitates delamination from the basal cell layer, a prerequisite for stratification [7]. Thus, loss of PKP4 expression in suprabasal epidermal cells promotes differentiation and stratification and hence contributes to epidermal homeostasis.

The regulation of the actin cytoskeleton and of cell cycle progression appears to be connected. Changes in actin filament structures and the filamentous/globular actin ratio influenced cell cycle progression and the disruption of actin filaments led to a G1 arrest [50, 51]. This actin-dependent arrest was linked to cyclin expression and cyclin-dependent kinase (CDK) activation [52]. Inhibition of ROCK activity in human keratinocytes increased proliferation [53]. This correlates with the reduced RhoA-ROCK activity and increased proliferation in PKP4 knockout keratinocytes and suggests that PKP4 might control proliferation through the regulation of ROCK activity. In support of a role of PKP4 in balancing proliferation and differentiation, PKP4 expression emerged as a favorable prognostic marker in renal and endometrial cancer (https://www.proteinatlas.org/ENSG00000144283-PKP4/pathology).

#### PKP4 in Hippo signaling

The evolutionarily conserved Hippo pathway plays an important role in organ size control and tissue homeostasis. The Hippo pathway comprises a kinase cascade with MST1/2 (mammalian ste2-like kinases 1/2) and the adaptor protein salvador (SAV1), which phosphorylate LATS1/2 (large tumor suppressor kinase 1/2) in complex with its regulatory protein MOB1 (mps one binder 1). MAP4K (mitogen-activated protein kinase kinase kinase kinase 1, MEKKK 1) is partially redundant with MST1/2 in the phosphorylation of LATS1/2 [54]. LATS1/2 phosphorylates and inactivates YAP1 and TAZ/WWTR. Phosphorylation of YAP1 by LATS1/2 interferes with its translocation into the nucleus [55, 56]. Phosphorylated YAP/TAZ either becomes degraded or is stabilized in the cytoplasm by 14-3-3 proteins, both preventing YAP/TAZ transcriptional activity. When Hippo signaling is inactive, dephosphorylated YAP/TAZ enters the nucleus, where it associates with TEAD transcription factors to promote target gene expression.

Cell junctions appear as major hubs that organize Hippo pathway components [57]. AJs can transduce mechanical stress into the Hippo pathway [58, 59]. At a molecular level, α-catenin was found in a tripartite complex with phosphorylated YAP and 14-3-3 in keratinocytes, which prevents YAP nuclear localization and inhibits its transcriptional activity [60, 61].

**Fig. 4:**
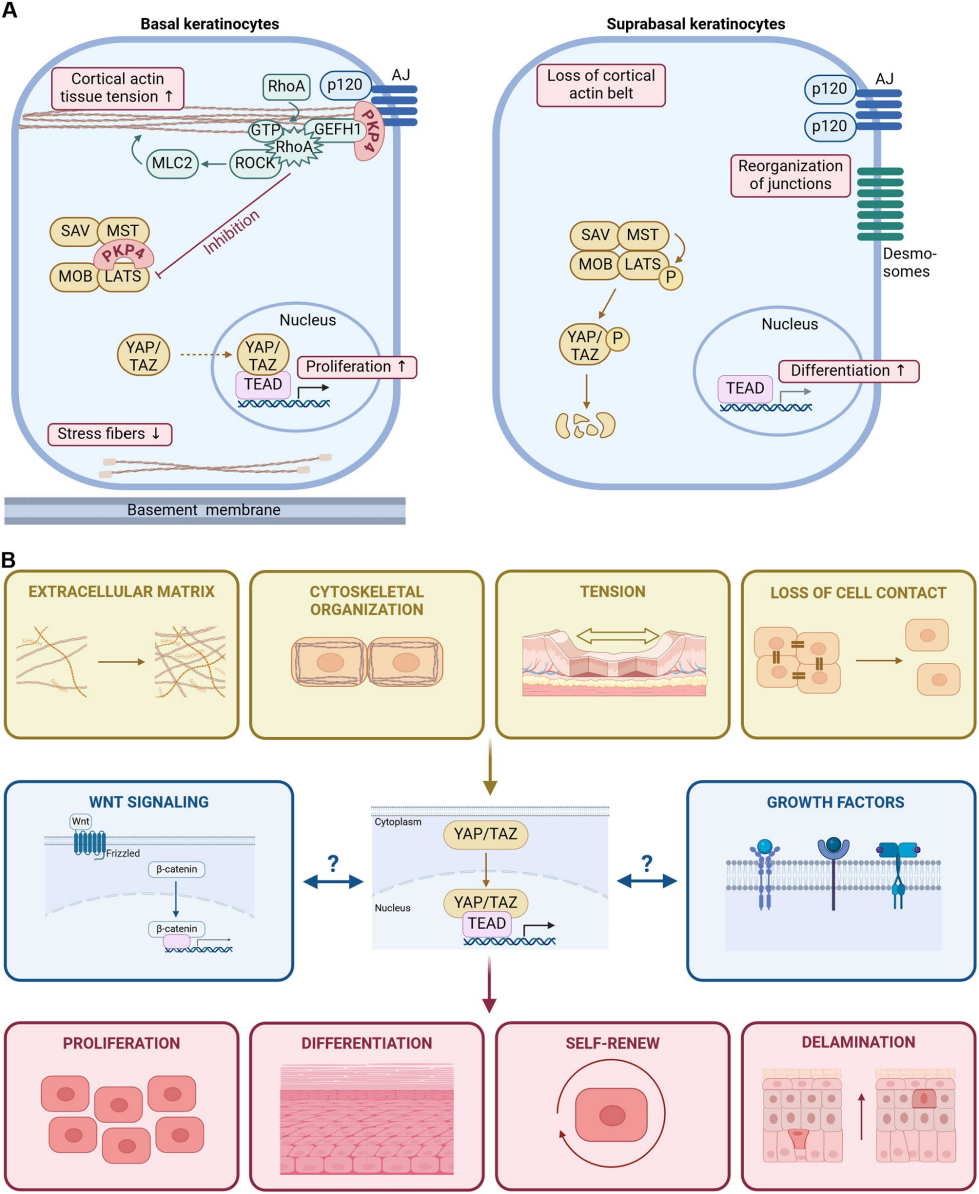
Role of PKP4 and Hippo signaling in epidermal keratinocytes. (**A**) Left panel: schematic depicting the roles of PKP4 in Rho- and Hippo signaling pathways in basal keratinocytes. PKP4 inhibits Hippo signaling. Inactive LATS promotes YAP/TAZ nuclear translocation and activation of YAP/TEAD target gene transcription that control cell proliferation. Right panel: suprabasal keratinocytes lack PKP4 expression. This leads to the activation of the Hippo pathway. The MST kinase phosphorylates LATS, which in turn phosphorylates YAP. Phosphorylated YAP becomes degraded. YAP/TEAD target genes are not expressed which facilitates differentiation. (**B**) Overview of YAP signaling regulators (upper row), interdependence of YAP signaling with other signaling pathways (middle row) and major effects of YAP signaling in the epidermis (lower row)

Rho- and Hippo signaling are interconnected: Rho-mediated mechanosignaling through the actin cytoskeleton promoted YAP/TEAD target gene transcription, while the Hippo pathway antagonizes YAP transcriptional activity through YAP phosphorylation, cytoplasmic retention and degradation [62]. Moreover, RhoA/ROCK/actomyosin inhibitors antagonized TEAD/YAP transcription while RhoA promoted YAP nuclear accumulation to increase TEAD/YAP target gene transcription (Fig. 4A) [63].

PKP4 is also involved in the regulation of Hippo signaling. PKP4 facilitated nuclear YAP/TAZ localization. Moreover, MST-, LATS- and YAP phosphorylation were considerably increased in PKP4-depleted cells and MAP4K, which can also phosphorylate LATS, was elevated [7, 64]. This resulted in YAP phosphorylation and the exclusion of phospho-YAP from the nucleus. Thus, PKP4 reduced YAP phosphorylation to facilitate its nuclear import and the transcriptional activation of YAP/TEAD target genes. Taken together, these data support a role of PKP4 in dampening Hippo signaling and activating YAP-mediated transcription of target genes involved in proliferation [7] (Fig. 4A). In support of a role of YAP in promoting keratinocyte proliferation, a conditional knockout of YAP in mouse skin caused epidermal hypoplasia due to insufficient proliferation [60] whereas the expression of a constitutive active YAP mutant (YAP-Ser127Ala) induced epidermal hyperthickening and impaired differentiation [60, 65]. Recent data indicate, that PKP4 dampens basal keratinocyte differentiation at least in part through YAP activation [7].

Rho signaling can inactivate LATS to prevent YAP phosphorylation resulting in YAP activation [66–68] (Fig. 4A). Accordingly, PKP4-mediated activation of RhoA would result in LATS inactivation and YAP activation in basal keratinocytes, indicating an indirect regulation of Hippo signaling by PKP4 (Fig. 4A). However, SAV1 and LATS1 as well as YAP/TAZ were co-isolated with PKP4, suggesting in addition a more direct role of PKP4 in YAP activation. Induction of keratinocyte differentiation by Ca^2+^ treatment increased PKP4-LATS association and translocated PKP4 together with LATS from the cytoplasm to cell junctions where LATS could phosphorylate YAP. Thus, during early steps of keratinocyte differentiation, while PKP4 is still present, recruitment of LATS and YAP to the junctional zone by PKP4 would activate the Hippo pathway to restrict nuclear localization and YAP/TEAD target gene expression and limit proliferation once the differentiation program has been initiated. YAP also promoted intercellular cohesion via PKP4. YAP depletion decreased the PKP4 protein level and reduced intercellular cohesion identifying YAP as a positive regulator of PKP4-mediated AJ function [7]. In a feedback loop, junctional PKP4 increased YAP association with the plasma membrane where YAP in turn seems to stabilize PKP4. Increased junctional PKP4 may not only strengthen cell-cell adhesion but in addition promote Rho-dependent signaling and tension formation at AJs thereby limiting LATS activity (Fig. 4A). This would in turn promote nuclear YAP localization and function. Thus, the PKP4-YAP connection can serve to precisely regulate intercellular adhesion and dynamics to enable cells to adapt to their chemical and mechanical environment.

#### The polycomb complex

Some evidence indicates that histone modifications can participate in mechanotransduction downstream of RhoA signaling [69, 70]. Histone modifications affect chromosome dynamics and gene expression by recruitment of non-histone proteins and altering chromatin structure. Histone modifications can directly regulate the expression of transcription factors and genes downstream of mechanical signaling pathways, and can also act as sensors of mechanical stimuli to feedback the activation or suppression of signaling pathways [71]. As mechanical transducers, YAP/TAZ can also regulate histone modifications. YAP functions have been linked to the Polycomb repressive complex. Polycomb comprises two protein complexes, Polycomb repressive complex 1 (PRC1) and PRC2. In the developing epidermis, disruption of either PRC1 or PRC2 resulted in the increased expression of a set of target genes [72] whereas the removal of both PRC1 and PRC2 provoked severe morphological defects [73–75]. A LATS2 knockout caused downregulation of PRC2 and H3K27me3. Thus, LATS2 promoted PRC2 histone methyltransferase activity and the expression of PRC2 components [76], thereby preventing premature differentiation. YAP also co-localized with enhancer of zeste homolog 2 (EZH2), the catalytically active subunit of PRC2, on the genome to repress transcription emphasizing an underappreciated aspect of YAP in transcriptional repression.

Well known mammalian target genes silenced by polycomb proteins include the *Hox* gene clusters [77]. YAP regulates the expression of Hoxa1 and Hoxc13 in in the epidermis [78]. Loss of PKP4 resulted in a remarkable upregulation of the *Hox* gene cluster suggesting a de-repression of polycomb targets in the absence of PKP4. Polycomb proteins are also master regulators that control the balance between proliferation and differentiation. When cells switch to terminal differentiation, the expression of differentiation genes is induced in part by removing the polycomb transcriptional repressor complex. Loss of polycomb function in epidermal precursor cells de-repressed genes relevant to epidermal differentiation, thereby accelerating the rate of maturation [75]. In line with the de-repression of polycomb target genes, PKP4 knockout cells revealed reduced expression of several polycomb group proteins including *Bmi1* and *Cbx* genes [7]. Whether the regulation of polycomb target genes through PKP4 is mediated through YAP or whether PKP4 is more directly involved in polycomb and histone regulation remains to be determined.

#### Wnt signaling

Wnts are secreted factors that bind to the frizzled family of seven transmembrane receptors. The canonical Wnt signaling pathway results in a β-catenin-dependent activation of TCF/LEF transcription factors thereby activating the transcription of target genes responsible for cellular proliferation and differentiation. The non-canonical β-catenin independent Wnt pathway triggers the activation of a RHOA-ROCK signaling axis leading to actin polymerization and reorganization [79]. How Wnt-dependent RhoA-ROCK signaling is interconnected with PKP4-mediated control of actin organization via RhoA-ROCK remains to be determined. Large scale sequencing of the transcriptome reveals that several frizzled receptors as well as several Wnt family members are downregulated in PKP4-knockout keratinocytes [7] suggesting a role of PKP4 in modulating Wnt signaling activity.

#### Growth factors

E-cadherin was shown to interact with and regulate the epidermal growth factor receptor (EGFR) indicative of a crosstalk between AJs and growth factor signaling [80, 81]. E-cadherin inhibited the activation of several receptor tyrosine kinases (RTKs) which was associated with decreased receptor mobility and ligand-binding affinity. This E-cadherin-dependent regulation of EGFR was induced by complex formation between EGFR and E-cadherin but was independent of β-catenin or p120ctn binding [81]. According to another report, E-cadherin inhibited EGFR signaling by a β-catenin-dependent mechanism [80]. Rübsam et al. [82] identified an E-cadherin-EGFR-driven mechanical signaling network in the epidermis, that spatiotemporally restricts intercellular tension to allow assembly of the TJ barrier only where it is spatially required. Junctional tension generated at AJs mediated EGFR localization and constrained its activity, which in turn was required for cortical reinforcement and subsequent formation of the TJ barrier [82]. Actomyosin-dependent forces also constrain EGFR activity in simple epithelia [83]. How the ubiquitously expressed E-cadherin controls localized EGFR activity is still an open question. High throughput screening for protein interactions identified the fibroblast growth factor receptor 2 (FGFR2) as a putative binding partner of PKP4 [84] raising the possibility that PKP4 contributes to the control of RTK activity. Moreover, several downstream targets of RTK signaling including RAS and RSK3 (https://thebiogrid.org/114074) [85] associated with PKP4 although these interactions have not been validated and their significance is unclear. Nevertheless, these data raise the possibility that PKP4 is also involved in modulating RTK-mediated signaling to adapt intercellular adhesion to environmental conditions.

## Conclusions and outlook

Epithelia need to be structurally robust to maintain a barrier to the outside environment. Intercellular cohesion mediated by AJs and desmosomes is essential for establishing such a barrier. PKP4 is a component of many but not all AJs. It stabilizes AJs by cadherin clustering and at the same time limits desmosome formation. This ensures stable but dynamic intercellular adhesion since AJs provide weaker cohesion compared to desmosomes. Regeneration of epithelia requires proliferation and in squamous epithelia delamination of cells from the basal layer. Cell shape changes during mitosis and delamination both require dynamic remodeling of junctions. In squamous epithelia such as the epidermis, these processes occur in the basal cell layer where PKP4 is part of AJs. PKP4-mediated recycling of cadherins ensures AJ dynamics, which is essential in the basal PKP4 expressing cell layer but nonessential in the suprabasal layers that lack PKP4 and provide a stable barrier mainly through desmosomal adhesion.

AJ regulate the organization of the underlying actin cytoskeleton and establish a hub for cell signaling. Recent data have uncovered a role of PKP4 in several signaling pathways. PKP4 plays a major role in the spatio-temporal regulation of Rho signaling, actin cytoskeleton organization and contractility. Thereby, PKP4 determines the mechanical properties of cells and controls junction maturation and stability. Rho signaling is interconnected with other signaling pathways. It controls the Hippo signaling pathway through inhibition of LATS, which allows YAP nuclear translocation and activation. Thus, PKP4 could modulate Hippo signaling through RhoA-mediated inhibition of LATS leading to Yap nuclear localization and activation which promotes proliferation. In addition, PKP4 seems more directly involved in Hippo signaling since it associates with several components of the pathway and affects their activity. Moreover, in a feedback mechanism YAP appears to stabilize PKP4-mediated intercellular adhesion. Thus, the functions of PKP4 in structurally stabilizing AJs as well as in signaling converge in the regulation of dynamic adhesion mediated by AJs. As a structural component of AJ or as a signaling platform PKP4 functions as a scaffold that brings proteins together either to cluster cadherins in AJs, to stabilize these structures, or to bring components of signaling cascades into close proximity to facilitate and enhance signal transduction at the right time and place. Since AJs are mechanosensitive structures, this enables cells to transduce mechanical signals into chemical signaling cascades. Figure 4B summarizes environmental conditions, signaling pathways as well as effects modulated by PKP4.

Numerous interacting proteins have been identified for PKP4 by large as well as small scale approaches (https://thebiogrid.org/114074) some of which are involved in the above mentioned functions. A major future challenge will be to elucidate how the diverse functions and interactions of PKP4 are regulated in the cell. PKP4 contains a vast number of phosphorylation sites on serine, threonine as well as tyrosine residues (83 serine, 36 threonine and 38 tyrosine residues, https://www.phosphosite.org/proteinAction.action?id=2590%26;showAllSites=true). Many sites were frequently found to be phosphorylated. Growth factor treatment with EGF, FGF1, FGF2, HGF and inhibitors such as gefitinib identified several tyrosine phosphorylation sites. Moreover, other modifications such as methylation, acetylation or palmitoylation may also play a role. To determine if and how these modifications determine PKP4’s localization and protein interactions is a major future challenge. Finally, it will be important to elucidate the interplay between PKP4 and the closely related catenins of AJs (p120ctn, ARVCF, δ-catenin) as well as the more distantly related desmosomal PKPs 1–3 to fully understand how these proteins modulate intercellular adhesion and signaling.

## Data Availability

No datasets were generated or analysed during the current study.

## Abbreviations

AJ: Adherens Junctions
ARHGAP23: Rho GTPase-Activating Protein 23
ARVCF: Armadillo Repeat gene deleted in Velo-Cardio-Facial syndrome
Cdc42: Cell division control protein 42
Ect2: Epithelial cell-transforming sequence 2
EGFR: Epidermal Growth Factor Receptor
EZH2: Enhancer of Zeste Homolog 2
FMRP: Fragile X Mental Retardation Protein
GAP: GTPase Activating Protein
GEF: Guanine Exchange Factor
KAP: Kinesin-associated protein 3
KIF: Kinesin-like protein
LATS: Large Tumor Suppressor kinase
LIMK: LIM-Kinase
MAP4K: Mitogen-Activated Protein Kinase kinase kinase kinase 1, MEKKK 1
MLC: Myosin Light Chain
MLCK: Myosin Light Chain Kinase
MOB: Mps One Binder kinase activator-like
MST: Mammalian Ste2-like kinase
Myo: Myosin
MYPT: Myosin Phosphatase-Targeting protein
NPRAP: Neural Plakophilin-Related Arm-Repeat Protein
PDZ: Postsynaptic density protein (PSD95), Drosophila disc large tumor suppressor (DlgA), and Zonula occludens-1 protein (zo-1)
PKP: Plakophilin
PRC1: Polycomb Repressive Complex 1
Rab: Ras-like proteins in brain
Rab11 Fip: Rab11 Family-interacting protein
RAC: Ras-related C3 botulinum toxin substrate
Rho: Ras homologue
ROCK: Rho-associated protein Kinase
RTK: Receptor Tyrosine Kinase
Sav: Salvador homolog
TAZ/WWTR1: Transcriptional coactivator with PDZ-binding motif/WW domain-containing Transcription Regulator protein 1
TCF/LEF: T-Cell Factor/Lymphoid Enhancer Factor
TEAD: TEA Domain Family Member
TJ: Tight Junction
Wnt: Wingless-related integration site
WT: Wildtype
YAP: Yes-Associated Protein
